# Fluid Overload-Associated Large B-Cell Lymphoma Presenting as Isolated Pleural Effusion

**DOI:** 10.3390/hematolrep18010013

**Published:** 2026-02-02

**Authors:** Kevin Leeper, Lauren Borecky, Mojtaba Akhtari, Jun Wang

**Affiliations:** 1 Department of Pathology and Laboratory Medicine, Loma Linda University Medical Center, Loma Linda, CA 92354, USA; 2 Division of Hematology and Oncology, Loma Linda University Medical Center, Loma Linda, CA 92354, USA

**Keywords:** fluid overload, large B-cell lymphoma, effusion, primary effusion lymphoma

## Abstract

Primary effusion-based lymphomas are uncommon and may pose significant diagnostic challenges. Fluid overload-associated large B-cell lymphoma is a recently recognized entity in the 5th edition of the World Health Organization Classification of Hematolymphoid Tumors and should be included in the differential diagnosis of effusion-based lymphomas, particularly in elderly immunocompetent patients with conditions that predispose to fluid overload. **Background and Clinical Significance:** We report a case of fluid overload-associated large B-cell lymphoma to add to the limited literature and highlight distinguishing features from other primary effusion lymphomas. **Case Presentation**: A 77-year-old male with end-stage renal disease on hemodialysis and heart failure with reduced ejection fraction was admitted for respiratory failure and found to have a right-sided pleural effusion. Two pleural fluid specimens examined several weeks apart revealed sheets of large atypical lymphoid cells positive for CD20, Pax-5, CD79a, CD45, MUM1, BCL2, BCL6 (weak) and negative for TTF1, CD68, MOC31, BER EP4, WT1, Calretinin, CD3, CD138, CD30, and cMYC. Human Herpesvirus-8 and Epstein–Barr virus were negative. Staging showed a few mildly fluorodeoxyglucose-avid mediastinal lymph nodes which were benign. Ultimately, the patient was diagnosed with fluid overload-associated large B-cell lymphoma and treated with rituximab, cyclophosphamide, vincristine sulfate, and prednisone, but passed away three months after diagnosis. **Conclusions**: Fluid overload-associated large B-cell lymphoma is a new and important diagnostic consideration in effusion-based lymphomas. It may be mistaken for other conditions such as primary effusion lymphoma or other diffuse large B-cell lymphomas. The presence of a Human Herpesvirus-8-negative effusion-based lymphoma in an elderly immunocompetent patient without nodal or tissue involvement should prompt consideration of fluid overload-associated large B-cell lymphoma.

## 1. Introduction and Clinical Significance

Fluid overload-associated large B-cell lymphoma (FO-LBCL) is a new entity in the 5th edition of the World Health Organization Classification of Hematolymphoid Tumors (WHO-HAEM5) [1]. The International Consensus Classification (ICC) describes a provisional entity termed “HHV-8 and Epstein–Barr virus–negative primary effusion-based lymphoma” [2,3]. Its key distinguishing criterion is Epstein–Barr virus (EBV) negativity, whereas WHO-HAEM5 reports that 13–30% of cases are EBV positive [1,2,3]. The ICC recommends expression of at least one B-cell marker to exclude plasmablastic neoplasms, while WHO-HAEM5 simply describes a mature B-cell immunophenotype, instead of plasmablastic [1,2,3]. To our knowledge, there are only 200 reported cases in the literature [4,5]. FO-LBCL presents in the eighth decade in patients with conditions that predispose to fluid overload, such as heart or kidney failure [1,5]. The patients present with symptoms related to effusions, most commonly in the pleural cavity, without nodal or soft tissue involvement [1,5,6]. The patients are immunocompetent and not infected with HHV8 [5,6,7]. Due to its low incidence and nonspecific presentation, FO-LBCL may face delays in diagnosis. For example, it may be mistaken for recurrent effusions secondary to heart failure exacerbation. It may be difficult to differentiate from similar diagnoses such as primary effusion lymphoma (PEL) or other diffuse large B-cell lymphomas (DLBCL) [1,7,8,9,10]. Some data suggests that FO-LBCL offers a favorable prognosis relative to diagnostic counterparts like PEL; therefore, accurate diagnosis is important [5,6]. We present the case of an elderly immunocompetent patient with heart failure with reduced ejection fraction and kidney failure who presented with respiratory failure and unilateral pleural effusion and was found to have FO-LBCL.

## 2. Case Presentation

A 77-year-old male with a history of chronic obstructive pulmonary disease, emphysema on chronic oxygen, end-stage renal disease on hemodialysis, heart failure with reduced ejection fraction (45–50%), coronary artery disease with four-vessel coronary artery bypass grafting, paroxysmal atrial fibrillation, and type II diabetes mellitus presented with worsening dyspnea following a missed dialysis appointment. The patient was admitted for respiratory failure due to volume overload and suspected pneumonia.

Of note, the patient had been hospitalized several times prior, including once at our institution, for similar volume overload symptoms. Unfortunately, due to the absence of shared medical records, the timing of these outside hospital admissions could not be reliably established. For clarity of the clinical timeline, significant events are laid out in Table 1.

Vitals on admission were significant for hypoxia requiring high-flow oxygen but were otherwise unremarkable. The complete blood count (CBC) with differential showed mild normocytic anemia and mild thrombocytopenia (Table 2). B-type natriuretic peptide (NT-proBNP) was elevated at 31,763.0 pg/mL (reference range < 450.0 pg/mL). A chest X-ray showed cardiomegaly with interstitial edema and moderate-sized right pleural effusion. Emergent hemodialysis was performed with 3.5 L of fluid removed, causing rapid improvement in oxygenation and volume status.

A thoracentesis was performed on the pleural effusion, and 2.5 L of exudative fluid was removed and sent for cytology and cultures; however, because no hematologic diagnosis was suspected at the time, no flow cytometry was performed. Cultures of the fluid were positive for *Pseudomonas aeruginosa* and extended spectrum beta lactamase positive *Escherichia* coli. The patient was placed on four weeks of intravenous meropenem.

**Figure 1:**
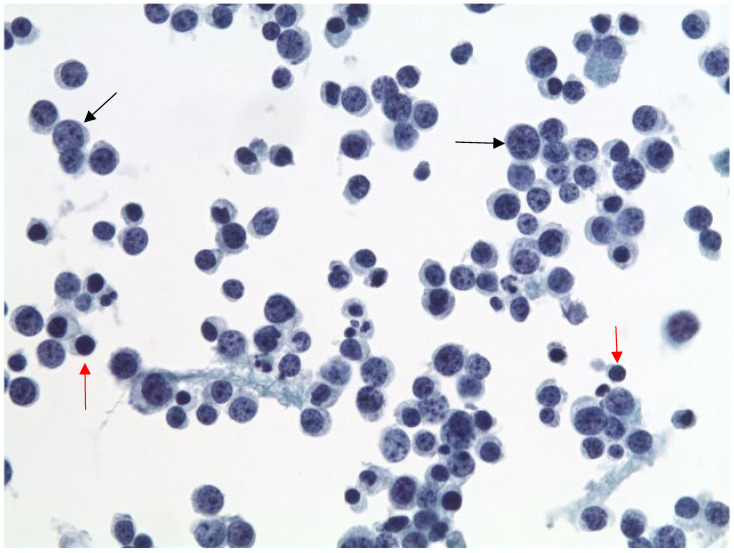
Pap, 400×. ThinPrep cytology reveals many large atypical lymphoid cells in a background of scattered small mature lymphocytes. The atypical cells show round to slightly irregular nuclear contours, open chromatin, and prominent nucleoli. Red arrows highlight background small, mature lymphocytes. Black arrows highlight large, atypical lymphoid cells.

**Figure 2:**
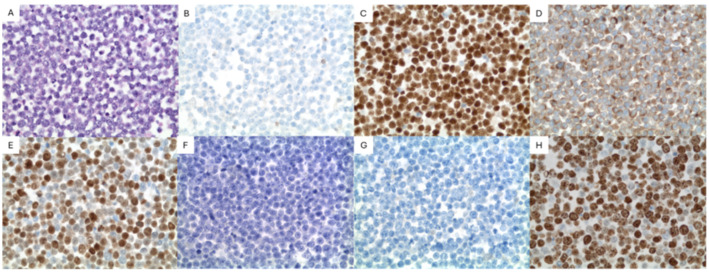
Cytopathologic and immunocytochemical features of second pleural fluid cell block specimen. (**A**) Hematoxylin and eosin (H&E), 400×. Cell block sections show sheets of large atypical lymphocytes with round to irregular nuclei, open chromatin and occasional conspicuous nucleoli. (**B**) CD3 negative in large atypical lymphocytes, 400×. (**C**) Pax-5 positive in large atypical lymphocytes, 400×. (**D**) BCL2 positive in large atypical lymphocytes, 400×. (**E**) MUM1 positive in large atypical lymphocytes, 400×. (**F**) HHV8 negative in large atypical lymphocytes, 400×. (**G**) CD138 negative in large atypical lymphocytes, 400×. (**H**) Ki-67 positive in large atypical lymphocytes (>95% overall), 400×. Due to similar appearance on H&E and immunocytochemistry, only photos of the second pleural fluid specimen are included.

Cytopathology of the right pleural fluid cell block revealed sheets of large atypical lymphoid cells (Figure 1). The immunocytochemistry of the large cells was positive for CD20, Pax-5, CD79a, CD45, MUM1, BCL6 (weak), and BCL2 and negative for TTF1, CD68, MOC31, BER EP4, WT1, Calretinin, CD3, CD138, CD30, and cMYC. Human herpes virus-8 (HHV8) and Epstein–Barr virus-encoded RNA (EBER-ISH) were negative (Figure 2). A high-grade B-cell lymphoma reflex fluorescence in situ hybridization (FISH) analysis for MYC (8q24) and MYC/IgH/CEN8 t(8;14) showed no abnormalities; therefore, BCL2 and BCL6 were not tested. The findings were signed out as positive for large B-cell lymphoma.

Further laboratory testing was completed following initial diagnosis and pertinent values are reported in Table 3. Notably, human immunodeficiency virus (HIV), hepatitis A (HAV), hepatitis B (HBV), and hepatitis C (HCV) were negative. Tumor lysis markers including lactate dehydrogenase were unremarkable.

Throughout an extended hospital course spanning nearly two months, the patient continued to develop right-sided pleural effusions which were treated with repeated chest tube placements. Meanwhile, the patient underwent extensive staging, including computed tomography (CT) of the chest with and without contrast, CT of the abdomen/pelvis with and without contrast, and CT of the soft tissue of neck with contrast. All of the performed CT imaging showed no masses or lymphadenopathy. A positron emission tomography (PET) scan showed mildly fluorodeoxyglucose-avid (FDG) mediastinal lymphadenopathy with max standardized uptake value (SUV) of 2.9. This was felt to be minimally suspicious for malignancy. The pleural fluid itself did not show significant FDG uptake. Cryobiopsy and transbronchial fine needle aspiration were performed on 5 of the mildly FDG-avid lymph nodes. All lymph node samples were benign, with only reactive changes.

Bone marrow trephine core and aspirate biopsy were performed and showed no evidence of marrow involvement by acute leukemia or lymphoma. Otherwise, the marrow was mildly hypercellular for age (~50% cellularity) with active trilineage hematopoiesis and mild mucinous degeneration of uncertain significance. Peripheral blood smear showed mild normocytic anemia and mild thrombocytopenia but was otherwise unremarkable.

Several weeks after the initial biopsy, another reaccumulation of the pleural effusion was drained and sent for pathology review. Still, the diagnosis of FO-LBCL had yet to be rendered. This time flow cytometry was performed and revealed a kappa monotypic B cell population (~58.8% of total events) expressing CD45, CD20, CD19, CD23 dim, FMC-7, CD38, CD43 dim, CD200 dim, and kappa light chain, but not CD5, CD10, CD11c, CD25, CD49d, CD103, CD123 and lambda light chain or other T cell markers tested. Cell block sections of the pleural fluid showed similar morphologic findings to the initial cytology; therefore, a less robust immunocytochemical panel was performed. Again, there were sheets of large atypical lymphoid cells. These cells were diffusely positive for PAX-5, BCL-2, and MUM1 and weakly positive for BCL-6. They were negative for CD3, CD5, CD10, CD30, CD138, CD38, and cMYC. The Ki-67 proliferation index showed strong positivity in >95% of cells. HHV8 and EBER ISH were again negative (Figure 2). FISH analysis for high-grade/large B-cell lymphoma detected a BCL6 (3q27) rearrangement (96%, cutoff 11.6%); however, it was negative for BCL2 (18q21) and MYC (8q24) rearrangement, thereby excluding double-hit lymphoma (Table 4). Ultimately, given the findings of an HHV8 negative large B-cell lymphoma on two separate biopsies with disease limited to the pleural cavity, a diagnosis of fluid overload-associated large B-cell lymphoma, non-germinal center subtype, was conferred.

Following the diagnosis, the patient was placed on rituximab with only minor side effects (several days of diarrhea). The anemia noted on admission was treated throughout the hospital course with Epoetin and intravenous Venofer. Prior to planned discharge, the patient again developed hypoxemia with perihilar edema on chest X-ray. A sputum culture grew methicillin-resistant *Staphylococcus aureus*. Following a week’s course of intravenous vancomyin, the patient was discharged to a long-term care facility for rehabilitation.

In the outpatient setting, the treatment regimen for the patient’s FO-LBCL was adjusted to rituximab, cyclophosphamide, vincristine sulfate, and prednisone (R-CVP). Rituximab, cyclophosphamide, hydroxydaunorubicin, oncovin, and prednisone (R-CHOP) were also considered; however, due to the patient’s end-stage renal failure, heart failure with reduced ejection fraction, and ECOG of 3, R-CVP was felt to be the most appropriate choice.

A week following discharge, the patient was again admitted for respiratory failure secondary to bilateral large malignant pleural effusions and suspected pneumonia. CT imaging of the cervical spine incidentally noted a C4 lamina and transverse process fracture which was managed non-operatively with a cervical collar. The patient was discharged once stabilized to baseline.

The patient passed away a month later at home, three months after initial diagnosis. Due to lack of medical documentation without our medical records, it is unclear whether this was directly a result of complications from FO-LBCL or from the many other comorbidities suffered by the patient.

## 3. Discussion

This case contributes to the limited literature on FO-LBCL, underscores diagnostic difficulties associated with the diagnosis, and highlights the key distinctions from primary effusion lymphoma and other similar-presenting large B-cell lymphomas.

The typical presentation of FO-LBCL is fairly non-specific. In our experience, even in the appropriate patient populations, most patients who present with recurrent effusions do not have a hematologic malignancy. And of those that do, most will not have FO-LBCL. Therefore, as seen in our case, clinical suspicion for FO-LBCL is often low. Beyond that, FO-LBCL is a new diagnosis, and physician awareness of the entity remains low. These factors contribute to diagnostic difficulty and were present in our case. To avoid a missed or delayed diagnosis, we suggest routine histopathologic sampling of recurrent effusions as well as maintaining a high index of suspicion when microscopically examining these specimens. Interestingly, patients who present with pericardial effusions may have better overall survival [5]. As hypothesized by Gisriel et al., this could be due to pericardial effusions causing the most severe symptoms, thereby prompting patients to seek more expedient care [5,11,12].

As mentioned previously, FO-LBCL exclusively involves the body cavities, most commonly the pleural cavity followed by the pericardial and abdominal cavities [1,4,5,6]. To our knowledge, only a single case of FO-LBCL with primary presentation outside the body cavities has ever been reported [13]. Consequently, staging must be performed to confirm a primary effusion-based lymphoma [1,4,5,6]. The presence of nodal or soft tissue involvement suggests secondary involvement of effusions by a primary large B-cell lymphoma [1,4,5,6]. In retrospect, the diagnosis of FO-LBCL likely could have been made once staging was complete, prior to the second thoracentesis. However, such a small delay likely had little, if any, impact on patient outcome.

Once a primary effusion-based lymphoma is confirmed, the differential diagnosis includes FO-LBCL, PEL and diffuse large B-cell lymphoma associated with chronic inflammation, also known as pyothorax-associated lymphoma (PAL). PAL can be distinguished by clinical history [14,15]. PAL occurs anywhere there is chronic inflammation, but most commonly involves the pleural cavity [1]. The typical PAL patient is a male in the seventh decade of life [15]. They have a history of pulmonary tuberculosis with decades of treatment via artificial pneumothorax [15]. The incidence of PAL has greatly decreased with the implementation of antibiotic treatment as artificial pneumothorax has long fallen out of favor [1,15]. This unique clinical presentation generally allows for distinction between PAL and other effusion-based lymphomas.

Immunohistochemical/immunocytochemical studies easily differentiate between FO-LBCL and PEL [1,4,5,6,14]. This case reflects the immunophenotype commonly seen in FO-LBCL. HHV8 infection, by definition, is not present in FO-LBCL. Conversely, HHV8 positivity is required to make a diagnosis of PEL [1]. Therefore, the presence or absence of HHV8 is the primary differentiating factor between FO-LBCL and PEL. EBV infection is present in only a small minority of FO-LBCL cases, approximately 10–15% [5,6,16]. Ki-67 proliferation index is usually high [5,6]. Most cases express pan B-cell markers with non-germinal center phenotype and are negative for plasma cell markers such as CD38 or CD138 [5,17]. To further aid differentiation, PEL typically expresses a plasma cell immunophenotype, with most cases also showing loss of pan B-cell marker expression [17,18]. Practically speaking, the presence of an HHV8-negative large B-cell lymphoma in an elderly immunocompetent patient’s effusion should prompt inclusion of FO-LBCL into the differential diagnosis. Subsequent staging can then be performed to confirm the diagnosis.

FISH analysis for BCL2, BCL6, and MYC rearrangements reveals rearrangements in only a small minority of cases (11%, 29% and 19%, respectively). In our case, BCL6 was rearranged (96%, cutoff 11.6%). Rearrangements in BCL2, MYC and t(8;14) were not present, excluding double-hit lymphoma.

Although not performed, polymerase chain reaction (PCR) reveals an IgH gene recombination in most cases [5,7]. As seen in Table 4, FISH testing for MYC/IgH/CEN8 t(8;14) revealed an abnormality described as “gain of IGH or IGH rearrangement” (76%, cutoff 16.2%). We speculate that this non-specific abnormality may represent a monoclonal IgH recombination, as commonly seen in FO-LBCL. In fact, the WHO-HAEM5 lists the presence of a clonal immunoglobulin gene recombination as a desirable diagnostic criterion for FO-LBCL [1].

The prognosis of FO-LBCL continues to evolve as additional cases are reported; however, current data suggests outcomes are generally more favorable than those of PEL and comparable to PAL [14,15]. Kaji et al. reported an 84.7% 2-year overall survival rate for FO-LBCL in a Japanese population [6]. A more recent review from Liu et al. found a 1-year overall survival rate of 47% in a Western population [13,14]. For comparison, PEL carries a 1-year overall survival rate of 30%, although prognosis is improving in the era of highly active antiretroviral therapy (HAART) [19]. Nakatsuka et al. reported 1- and 3- year survival rates of 48.6% and 27.0%, respectively, for PAL [15]. Although our patient survived only three months after diagnosis, this interval likely does not reflect his true overall disease duration. The initial history and physical noted previous admissions at an outside hospital for recurrent effusions; however, the absence of documented dates and unavailable outside medical records limits precise disease timeline reconstruction. Therefore, it is probable that the patient developed FO-LBCL prior to his first admission at our institution. Unfortunately, we cannot reliably identify the exact onset, which precludes determination of a more accurate survival timeframe.

It is also important to recognize that the comorbidities predisposing patients to FO-LBCL carry substantial inherent morbidity and mortality. Poor patient performance status, existing severe comorbidities or personal preference may affect treatment choice, for example, thereby lowering overall survival. In other words, although the disease-related mortality of FO-LBCL is felt to be “favorable” relative to other entities such as PEL, a patient’s overall prognosis may still be extremely poor due to comorbidity-related mortality. Unfortunately, due to unavailability of external medical records, we are unable to determine whether our patient’s death was largely disease-related versus comorbidity-related.

Although no standardized regimen exists for FO-LBCL, most reported cases used CHOP or R-CHOP in conjunction with drainage of effusions [3,4,11]. In cases such as ours, patients with severe comorbidities may not be good candidates for CHOP or R-CHOP. For these patients, alternative therapies, such as R-CVP in our case, can be considered. Some cases are managed with therapeutic drainage of effusions alone [3]. Rare, published cases have reported success using intra-pleural rituximab to treat CD20+ non-Hodgkin lymphomas causing recurrent pleural effusions, with minimal to no systemic side effectcs [20,21]. Given that the majority of FO-LBCL cases express CD20, intrapleural rituximab may represent a reasonable therapeutic alternative for patients unable to tolerate systemic therapy or those with persistent effusions despite systemic treatment.

In conclusion, the presented case demonstrates the importance of considering FO-LBCL when faced with an effusion-based lymphoma. Although a comprehensive work-up, including immunophenotyping, ancillary testing, and clinical imaging, is required, the diagnosis is relatively straightforward once completed. Prompt and accurate diagnosis is especially important, as FO-LBCL may offer a more favorable diagnosis relative to other diagnostic considerations like PEL [3,4]. Furthermore, we contribute to the limited published literature on FO-LBCL.

## Figures and Tables

**Table 1:** Summary of major clinical events leading to the diagnosis of FO-LBCL.

Dates	Clinical Event
26 July 2025	First in-house documentation of right pleural effusion
15 August–9 October 2025	Hospital admission with volume-overload related symptoms and suspected pneumonia
23 August 2025	First thoracentesis performed
29 August 2025 17 September 2025	Initial pathologic diagnosis rendered: “Suspicious for large B-cell lymphoma” Staging fully completed including lymph node biopsy, bone marrow biopsy, and imaging
19 September 2025	Second thoracentesis performed
26 September 2025	Pathologic diagnosis refined to “High-grade large B-cell lymphoma, non–germinal center subtype”
27 September 2025 10 October 2025	Following literature review and clinical, radiologic, and pathologic correlation, FO-LBCL enters differential diagnosis and the patient is formally diagnosed Patient passed away

**Table 2:** Complete blood count with differential upon admission.

Reference Range and Units	Values
WBC 4.80–11.80 bil/L	5.65
RBC 3.80–5.30 tril/L	3.71
Hgb 33.5–47.0% g/dL	10.4
Hct 33.5–47.0%	32.1
MCV 77.0–9.6 fL	86.5
MCH 24.5–32.0 pg	28.0
MCHC 30.0–35.0 g/dL	32.4
RDW 12.0–15.0%	19.7
Plts 140–340	Clumping present, estimated ~80
MPV 0.0–15.0	9.45; possible large plts present
nRBC, absolute (abs) ≤0.01 bil/L	<0.01
ANC 2.20–9.20 bil/L	4.57
Neutrophil % 45.0–85.0%	80.8
Lymphocyte % 7.0–40.0%	8.5
Lymphocytes 0.30–4.70 bil/L	0.40
Monocyte 0.00–1.20 bil/L	0.52
Monocyte % 2.0–10.0%	9.2
Eosinophil 0.00–0.80 bil/L	<0.03
Eosinophil % 0.00–6.5%	0.4
Basophil 0.00–0.50 bil/L	<0.03
Basophil % 0.0–1.2%	0.4
Immature granulocytes, % 0.0–0.5%	0.7
Immature granulocytes, abs 0.00–0.02 bil/L	0.04

**Table 3:** Pertinent laboratory testing completed after initial diagnosis.

Reference Range and Units	Values
HIV antigen, antibody	Non-reactive
Hepatitis A antibody IgM acute	Non-reactive
Hepatitis B core antibody	Non-reactive
Hepatitis C antibody screen	Non-reactive
Lactate dehydrogenase (LDH) 120–270 U/L	194
Fibrinogen 200–393 mg/dL	505
Uric acid 3.5–8.3 mg/dL	4.3
Prothrombin time (PT) 9.4–12.5 s	12.5
International normalized ratio (INR) 0.8–1.1	1.1
Partial thromboplastin time (PTT)	36.3
Iron 65–175 ug/dL	47
Ferritin 12.0–350.0 ng/mL	289
Folate 3.5–17.5 ng/mL	7.3
Vitamin B12 200.0–1240.0 pg/mL	1305

**Table 4:** FISH analysis for high-grade/large B-cell lymphoma showing a BCL6 rearrangement in 96% of cells (cutoff 11.6%).

	Interpretation	Signal Pattern	Abnormality Identified	%	Cutoff
BCL2 (18q21) Rearrangement	Not Detected (Atypical)	>2F	Gains (trisomy/tetrasomy)	96.0%	16.2%
BCL6 (3q27) Rearrangement	Detected	≥1R ≥ 1G ≥ 1F	BCL6 gene rearrangement	96.0%	11.6%
MYC (8q24) Rearrangement	Not Detected (Atypical)	3F to 4F	MYC gains (trisomy/tetrasomy)	72.0%	16.2%
MYC/IgH/CEN8 t(8;14)	Not Detected (Atypical)	3~4R > 2G3~4A	Gains of MYC/chromosome 8 Gain of IGH/chromosome 14/14q or IGH rearrangement (not to MYC)	76.0%	16.2%

## Data Availability

The data presented in this study are only available on request from the corresponding author due to patient privacy.

## Abbreviations

The following abbreviations are used in this manuscript:

RNA	Ribonucleic acid
ECOG	Eastern Cooperative Oncology Group performance status
IgH	Immunoglobulin heavy chain
WBC	White blood cells
RBC	Red blood cells
Hgb	Hemoglobin
Hct	Hematocrit
MCV	Mean corpuscular volume
MCH	Mean corpuscular hemoglobin
MCHC	Mean corpuscular hemoglobin concentration
RDW	Red cell distribution width
Plts	Platelets
MPV	Mean platelet volume
nRBC	Nucleated red blood cells
ANC	Absolute neutrophil count
bil	Billion
L	Liter
bil/L	Billions per liter
tril/L	Trillions per liter
Tril	Trillion
pg	Picograms
pg/mL	Picograms per milliliter
g	Gram
mL	Milliliter
dL	Deciliter
g/dL	Grams per deciliter
mg/dL	Milligrams per deciliter
fL	Femtoliter
U/L	Units per liter
ng/mL	Nanograms per milliliter
ug/dL	Micrograms per deciliter

## References

[1] Ott G., Siebert R., Alaggio R., de Jong D., Naresh K., Dave S., Coupland S. (2024). Chapter 4: B-cell lymphoid proliferations and lymphomas. Large B-cell lymphomas. Haematolymphoid Tumours.

[2] Di Napoli A., Soma L., Quintanilla-Martinez L., de Leval L., Leoncini L., Zamò A., Ng S.-B., Ondrejka S.L., Climent F., Wotherspoon A. (2023). Cavity-based lymphomas: Challenges and novel concepts. A report of the 2022 EA4HP/SH lymphoma workshop. Virchows Arch..

[3] Campo E., Jaffe E.S., Cook J.R., Quintanilla-Martinez L., Swerdlow S.H., Anderson K.C., Brousset P., Cerroni L., de Leval L., Dirnhofer S. (2022). The International Consensus Classification of Mature Lymphoid Neoplasms: A report from the Clinical Advisory Committee. Blood.

[4] Graham C., Gupta S., Haddad P.A. (2023). Fluid-Overload Associated Large B-Cell Lymphoma Clinicopathologic Features and Determinants of Outcomes: Analysis of a Pooled Database. Blood.

[5] Gisriel S.D., Yuan J., Braunberger R.C., Maracaja D.L., Chen X., Wu X., McCracken J., Chen M., Xie Y., Brown L.E. (2022). Human herpesvirus 8-negative effusion-based large B-cell lymphoma: A distinct entity with unique clinicopathologic characteristics. Mod. Pathol..

[6] Kaji D., Ota Y., Sato Y., Nagafuji K., Ueda Y., Okamoto M., Terasaki Y., Tsuyama N., Matsue K., Kinoshita T. (2020). Primary human herpesvirus 8-negative effusion-based lymphoma: A large B-cell lymphoma with favorable prognosis. Blood Adv..

[7] Alexanian S., Said J., Lones M., Pullarkat S.T. (2013). KSHV/HHV8-negative effusion-based lymphoma, a distinct entity associated with fluid overload states. Am. J. Surg. Pathol..

[8] Bahmad H.F., Gomez A.S., Deb A., Safdie F.M., Sriganeshan V. (2023). Fluid Overload-Associated Large B-Cell Lymphoma: A Case Report and Review of Literature. Hematol. Rep..

[9] Koh J., Shin S.A., Lee J.A., Jeon Y.K. (2022). Lymphoproliferative disorder involving body fluid: Diagnostic approaches and roles of ancillary studies. J. Pathol. Transl. Med..

[10] Kim M., An J., Yoon S.O., Yong S.H., Kim J.S., Yang W.I., Leem A.Y. (2020). Human herpesvirus 8-negative effusion-based lymphoma with indolent clinical behavior in an elderly patient: A case report and literature review. Oncol. Lett..

[11] Sun S., Li T., Liu Y., Wu M., Zhao Q., Kang N., Chen J., Chen H., Song J. (2025). Massive Pericardial Effusion as the Initial and Main Manifestation of Fluid Overload-Associated Large B-Cell Lymphoma. JACC Case Rep..

[12] Pascual L.P., Crespo F.J.D., López B.B.A., de Los Ángeles Pérez Saénz M., Alonso R.M., Pinilla S.M.R., de la Pinta F.J.D. (2025). Large B-cell lymphoma associated with fluid overload: Two new case reports with molecular insights. Virchows Arch..

[13] Wu W., Youm W., Rezk S.A., Zhao X. (2013). Human herpesvirus 8-unrelated primary effusion lymphoma-like lymphoma: Report of a rare case and review of 54 cases in the literature. Am. J. Clin. Pathol..

[14] Liu C.Y., Chen B.J., Chuang S.S. (2022). Primary Effusion Lymphoma: A Timely Review on the Association with HIV, HHV8, and EBV. Diagnostics.

[15] Nakatsuka S., Yao M., Hoshida Y., Yamamoto S., Iuchi K., Aozasa K. (2002). Pyothorax-associated lymphoma: A review of 106 cases. J. Clin. Oncol..

[16] Usmani A., Walts A.E., Patel S., Alkan S., Kitahara S. (2015). HHV8-negative effusion based lymphoma: A series of 17 cases at a single institution. J. Am. Soc. Cytopathol..

[17] Gathers D.A., Galloway E., Kelemen K., Rosenthal A., Gibson S.E., Munoz J. (2022). Primary Effusion Lymphoma: A Clinicopathologic Perspective. Cancers.

[18] Chen B.J., Wang R.C., Ho C.H., Yuan C.-T., Huang W.-T., Yang S.-F., Hsieh P.-P., Yung Y.-C., Lin S.-Y., Hsu C.-F. (2018). Primary effusion lymphoma in Taiwan shows two distinctive clinicopathological subtypes with rare human immunodeficiency virus association. Histopathology.

[19] El-Fattah M.A. (2017). Clinical characteristics and survival outcome of primary effusion lymphoma: A review of 105 patients. Hematol. Oncol..

[20] Rivas-Vera S., Cadena-Euman C., Chalapud-Revelo J., Aguilar-Navarro A., Sobrevilla-Calvo P., Lievano-Torres C. (2008). Intrapleural Rituximab (R) Is Effective in Refractory Pleural Effusion Due to Non- Hodgkin’s Lymphoma. Blood.

[21] Schmidt H.H., Renner H., Linkesch W. (2004). Intrapleural instillation of rituximab for the treatment of malignant pleural effusions in NHL. Haematologica.

